# Effects of Ropivacaine in Patient-Controlled Epidural Analgesia on Uterine Electromyographic Activities during Labor

**DOI:** 10.1155/2018/7162865

**Published:** 2018-11-07

**Authors:** Xueya Qian, Qingning Wang, Xinxu Ou, Pin Li, Baisong Zhao, Huishu Liu

**Affiliations:** ^1^Department of Obstetrics, Guangzhou Women and Children's Medical Center, Guangzhou Medical University, Guangzhou, China; ^2^Department of Anesthesia, Guangzhou Women and Children's Medical Center, Guangzhou Medical University, Guangzhou, China

## Abstract

Epidural analgesia is effective in relieving pain during labor. However, concerns as to compromised labor progress and outcomes arise. This study aimed to assess the effect of patient-controlled epidural analgesia (PCEA) with ropivacaine on uterine electromyography (EMG) activities and outcomes in labor. A total of 213 pregnant women were divided into three groups: the PCEA with ropivacaine group (n = 78), the PCEA with levobupivacaine group (n = 66), and a control group that did not receive PCEA (n = 69). Uterine EMG activities were recorded during the first stage of labor. Maternal and fetal outcomes also were assessed. The primary outcomes of this study were EMG activities. No significant differences were observed in patient demographics or neonatal weight among the three groups. Compared to the PCEA with levobupivacaine group, the control and PCEA with ropivacaine groups had lower rates of oxytocin administration (P < 0.05) and shorter durations of the first stage of labor (P < 0.05). For the EMG activities, the PCEA with ropivacaine group showed a higher power (P < 0.01) and higher peak frequency (P < 0.05) than the PCEA with levobupivacaine group. With ropivacaine, the EMG activities remained stable 30–120 min. Compared with levobupivacaine, the use of ropivacaine in PCEA has no suppressive effect on uterine EMG activities during the first stage of labor. In addition, ropivacaine leads to labor progress and delivery outcomes similar to those in the control group, as well as similar and favorable analgesic satisfaction with the use of levobupivacaine.

## 1. Introduction

Patient-controlled epidural analgesia (PCEA) is a well-accepted technique for pain relief during labor. However, there are still concerns that epidural labor analgesia may lead to prolongation of labor [1, 2], malposition of the fetal head [3], increased use of oxytocin [4, 5], and even increased instrumental deliveries [5, 6]. These effects may be due to the direct inhibition of the myometrial contractions by local anesthetics during labor [7–9].

Ropivacaine has been used as obstetric anesthesia because it offers good analgesic properties without causing significant motor blockade or systemic toxicity [10–12]. However, whether the use of ropivacaine affects uterine contractions is largely unknown. An important reason may be the lack of an objective and precise method for evaluating the effect of PCEA on myometrial contraction during labor. Recently, uterine electromyography (EMG) has emerged as a useful method for monitoring the excitability and contractility of the myometrium [13–15]. Our previous study indicated that uterine EMG was valuable for assessing uterine muscle activities during labor [16]. We also found that PCEA with levobupivacaine suppressed uterine EMG activities and prolonged the first stage of labor [17]. Considering the advantage of ropivacaine without significant motor blockade during analgesia, we hypothesized that PCEA with ropivacaine could have less inhibitory effects on uterine EMG activities compared to levobupivacaine during labor. The objective of this study was to investigate uterine EMG activities and labor outcomes in patients receiving PCEA with ropivacaine.

## 2. Material and Methods

### 2.1. Patients

This prospective cohort study included 213 patients at the Guangzhou Women and Children's Medical Center treated between 2015 and 2018. The study protocol was approved by the institutional review board (protocol No. 2014110533) and registered at ClinicalTrials.gov (registration No. NCT02036242). Patients were included in this study if they met the following inclusion criteria: (1) singleton pregnancy, age ≤ 35 years, and a gestational age of 37–42 weeks; and (2) fetus with cephalic presentation. According to the standard obstetric indications, oxytocin was titrated by the obstetricians. The completed gestational weeks were determined by the date of the first day of the last menstrual period and confirmed by an ultrasound scan. Women with medical complications of pregnancy that required delivery via cesarean section or interventions that could influence the labor duration were excluded.

Patients were divided into three groups: those who received PCEA with levobupivacaine (levobupivacaine group, n=66), those who received PCEA with ropivacaine (ropivacaine group, n=78), and those who did not receive PCEA (control group, n=69) nor other types of analgesia. Written informed consent was obtained from all patients. In our practice, whether or not PCEA treatment was administered was based on the patient's choice, and the use of levobupivacaine or ropivacaine was decided by the anesthesiologists.

According to the different time points of EMG recording, patients receiving PCEA with ropivacaine were subdivided into three groups: 30 min (n = 26), 60 min (n = 27), and 120 min (n = 25).

### 2.2. PCEA Protocol

After epidural catheter placement into the L2–L3 interspace, patients in the two PCEA groups received a first analgesic drug of 0.0625% ropivacaine and 0.0625% levobupivacaine in 10 ml saline, as well as 5 *μ*g sufentanil. Patients were then placed in the supine position with left uterine displacement. A PCEA device containing 0.0625% ropivacaine or levobupivacaine and 0.4 *μ*g/ml sufentanil in 240 ml saline was started, with a background infusion rate of 6 ml/h and a bolus dose rate of 8 ml for a lockout time of 15 min. An anesthesiologist who was unaware of the grouping condition scored and recorded the visual analog scale (VAS) score (0–10) for pain reported by the patients at 0, 15, and 30 min after PCEA catheterization. Oxytocin was only administered after labor if needed. Patient characteristics including age, body mass index (BMI), gestational age, and oxytocin administration were recorded. Maternal and fetal outcomes including neonatal weight, duration of the first stage of labor, blood loss within 2 h after placenta delivery, Apgar scores at 1, 5, and 10 min, and incidence of meconium-stained amniotic fluid were also recorded.

### 2.3. EMG Recording

Uterine EMG data were collected noninvasively from the abdominal surface using the PowerLab electromyographic instrumentation (AD Instruments, Castle Hill, Australia). In detail, four Ag-AgCl Beckman differential bipolar electrodes (Jun Kang Medical Supplies, Shanghai, China) were placed around the navel, with each electrode separated from the other by a distance of 3 cm. A reference electrode was attached on the hip of the patient.

A specific filter with a band-pass of 0.34–1.00 Hz was used to exclude interference signal components during EMG recording [18–20]. Using an external tocodynamometry (TOCO, Sunray, Guangzhou, China) connected with the PowerLab and a standard maternal-fetal monitor (Philips, Avalon FM20, Best, The Netherlands), patients were continuously monitored for 30 min. EMG activity recordings included the duration of burst, number of burst, root mean square (RMS), power, and peak frequency.

### 2.4. EMG Analysis

As described in our previous studies, standardized criteria were used to identify uterine EMG bursts: (1) a set of positive signals with an amplitude twice the baseline values; (2) a set of signals not returning to the baseline within 15 seconds; and (3) a burst often accompanied by contractions displayed on the TOCO [16, 21]. To assess the signal stability, uterine EMG bursts were analyzed 10 min after the beginning of the recording as a standard procedure. One trained investigator who was experienced in EMG procedures performed the EMG and analyzed the data using Chart 8.0 software (ADInstruments, Castle Hill, Australia).

### 2.5. Statistical Analysis

Statistical analyses were performed using SPSS software (version 22.0; IBM SPSS Inc., Chicago, IL). On sample size calculations, 47 subjects per group were required with a power of 0.90 and an alpha of 0.05 based on our previous studies [16, 21]. Continuous variables were compared using one-way analysis of variance or Kruskal-Wallis test when appropriate for pairwise comparisons and because of non-normal distribution of variables. Categorical variables were analyzed using the Chi-squared test. Two-sided P-values < 0.05 were considered statistically significant.

## 3. Results

### 3.1. Patient Characteristics and Labor Outcomes

As shown in Table 1, patients in the three groups had similar demographic characteristics. The percentages of patients who needed oxytocin administration after labor were significantly lower in the control and ropivacaine groups compared to the levobupivacaine group (P=0.02).

Table 2 describes the maternal and fetal outcomes. The duration of the first stage of labor was significantly shorter in the control and ropivacaine groups than in the levobupivacaine group. Patients in the ropivacaine group had higher Apgar scores at 1 and 5 min (P<0.001) compared to those in the other two groups. All patients in the three groups had similar labor outcomes including cesarean delivery rate, instrumental delivery rate, and postpartum hemorrhage.

### 3.2. Comparison of Analgesic Effects

**Figure 1:**
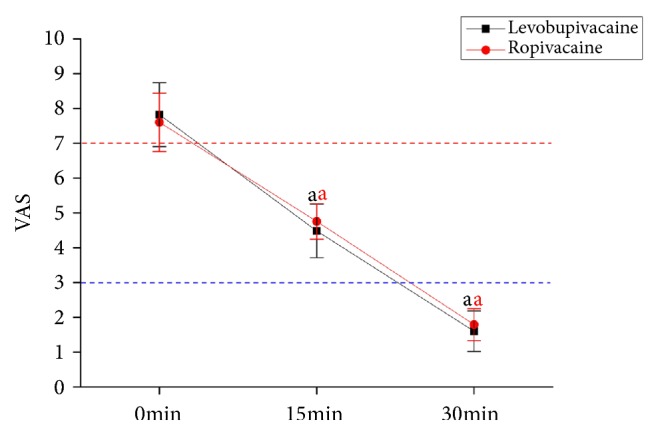
**Analgesic effects of levobupivacaine and ropivacaine.** VAS: visual analog scale for pain. The VAS score did not differ statistically between the two groups at the same time points. The horizontal red and blue lines indicate 7 and 3 on the VAS scale, respectively (7–10 for severe pain, 4–6 for moderate pain, and 0–3 for mild pain).

As shown in Figure 1, patients in both groups were in severe pain at the time when labor analgesia was administered. The VAS scores of the two groups decreased significantly over time (P<0.05) but did not differ significantly between the two groups at the same time point (P>0.05). It was suggested that when levobupivacaine and ropivacaine were used in PCEA, they both took effect within 15 minutes and achieved a good analgesic effect within 30 minutes. Consistently, no difference was observed in the analgesic effect of the two drugs.

### 3.3. EMG Activities

**Figure 2:**
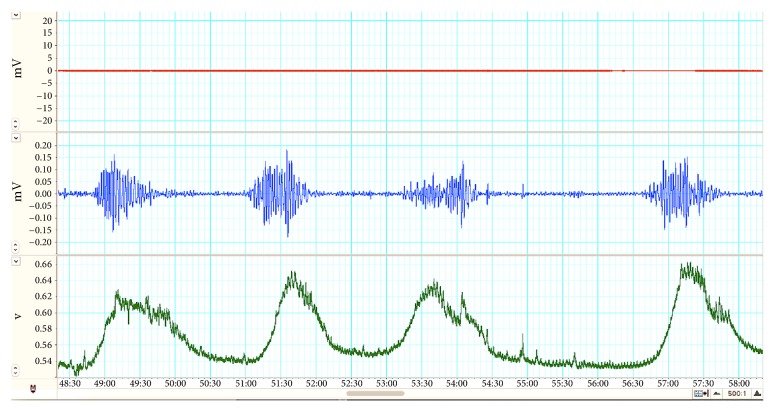
**Representative EMG images.** The representative recordings of ropivacaine group during the 1st stage of labor showing EMG signals from abdominal muscle (top tracings in red), uterine (middle tracings in blue), and TOCO signals (bottom tracings in green). A horizontal line above some of the bursts denotes bursts. The TOCO recorded signals (green tracings) correspond to TOCO recorded uterine contractions and occur at about the same time as EMG bursts.

**Figure 3:**
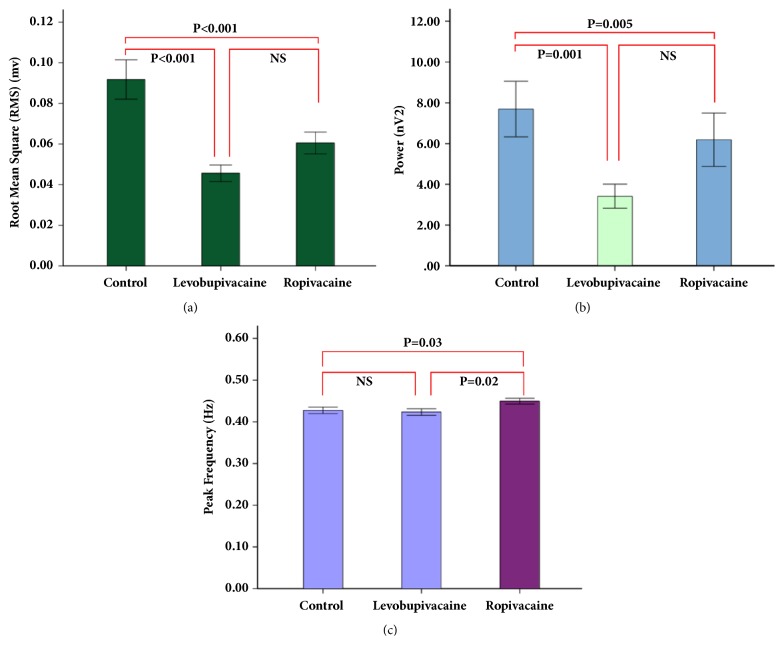
**RMS, power, and peak frequency of EMG in the three groups.** (a) RMS. (b) Power. (c) Peak frequency.

Representative EMG image of the patient in ropivacaine group is shown in Figure 2. The results for uterine EMG activities are shown in Table 3. The two PECA groups had a significantly lower RMS compared to the control group (P < 0.001; Figure 3(a)). Both the control and ropivacaine groups had a higher power than the levobupivacaine group (P = 0.005; Figure 3(b)). The peak frequency was significantly higher in the ropivacaine group than in either of the other two groups (Figure 3(c)). There were no significant differences in the duration or the number of bursts among the three groups (P>0.05).

Regarding the EMG results at 30, 60, and 90 min in the ropivacaine group, there was no significant difference in uterine EMG activity among the three different time points (Table 4).

## 4. Discussion

In this study, we assessed uterine EMG activities and obstetric outcomes during the first stage of labor with different labor analgesia regimens. The results suggest that (1) ropivacaine use had no inhibitory effect on uterine contractions compared with levobupivacaine; (2) ropivacaine use did not prolong the labor progress or impact delivery outcomes; and (3) ropivacaine use produced a comparable analgesic effect to that of levobupivacaine for epidural labor analgesia.

In 1984, bupivacaine (0.75% solution) was reported to cause a number of obstetric deaths due to its cardiotoxicity. Then, the search for a safe as well as long-acting local anesthetic was launched. Two L-isomeric anesthetic agents, ropivacaine and levobupivacaine, have been developed. These two local anesthetics are better alternatives for epidural labor analgesia with less cardiac and neurological toxicity compared to bupivacaine [22, 23].

Ropivacaine is less likely to cause motor blockade and neurotoxicity due to its relatively low lipophilic capacity and resistance to penetrating readily into the myelinated nerve fibers [24, 25]. However, the superiority of ropivacaine over levobupivacaine for epidural labor analgesia remains controversial. Several studies indicated that ropivacaine and levobupivacaine have similar sensory and motor blocking effects in epidural labor analgesia [26–28], while another study showed that ropivacaine produced a similar level of analgesia as levobupivacaine but a significantly lower level of motor block [29]. In experimental studies, controversies regarding the effects of local anesthetics on myometrial contractility also exist [30–32]. In our study, the VAS pain scores of the ropivacaine group were comparable to those of the levobupivacaine group. Both levobupivacaine and ropivacaine, with a low concentration of 0.0625%, produced good analgesic effects. These effects were mainly due to the use of a pulsed electronic infusion pump in the PCEA protocol through which the local anesthetics were administered and tailored by each individual patient.

Regarding uterine EMG activities, the values of RMS and power were significant higher in the ropivacaine group than in the levobupivacaine group. In addition, uterine EMG activities in the ropivacaine group were similar to those in the control group. Consistent with the findings on EMG, patients in the ropivacaine and control groups had a similar duration of the first stage of labor, while those in the levobupivacaine group had a significantly longer labor duration. These results were also consistent with our previous finding that levobupivacaine suppressed uterine EMG and prolonged the first stage of labor [17]. Moreover, the need for oxytocin administration was lower in the ropivacaine group compared with the levobupivacaine group. At different time points from 30 to 120 min, the uterine EMG activity did not differ significantly in the ropivacaine group compared with the other groups. Taken together, the use of ropivacaine resulted in a less inhibitory effect on uterine contractility and labor progress than levobupivacaine.

There are several limitations to this study. First is the study design. This is a prospective cohort study rather than a randomized trial, and thus a potential bias may exist. Second, as previously reported, deep motor blockade caused by local anesthetics also prolongs the second stage of labor by reducing voluntary maternal expulsive forces [24]. However, in this study, we only investigated the EMG activities during the first stage. We did not evaluate the motor block effect of ropivacaine in the second stage of labor too. Another limitation was the lack of pain assessment after 30 min. Further prospective studies are needed to strengthen these findings and the benefits of ropivacaine in epidural labor analgesia.

## 5. Conclusions

In conclusion, this is the first study to demonstrate that PCEA with ropivacaine leads to a better analgesic satisfaction without adverse effects on uterine EMG activities or obstetric outcomes during the first stage of labor compared to levobupivacaine. To obtain the best epidural anesthetic effect and obstetric outcomes without adverse effects, the optimal PCEA regimen for pain relief during labor requires further investigations.

## Figures and Tables

**Table 1:** Patient characteristics.

	**Control**	**Levobupivacaine**	**Ropivacaine**	**P value**
	**n = 69**	**n = 66**	**n = 78**	
**Age (years)**	27.99 ± 0.38	28.74 ± 0.36	28.94 ± 0.36	0.16
**BMI (kg/m** ^**2**^ **)**	25.23 ± 0.31	25.27 ± 0.28	26.17 ± 0.40	0.09
**Gestational age (weeks)**	39.40 ± 0.13	39.69 ± 0.12	39.58 ± 0.11	0.23
**Multipara, n (**%**)**	7 (10.15%)	9 (13.64%)	8 (10.26%)	0.77
**Oxytocin administration, n (**%**)**	18 (26.09%)^a^	31 (46.97%)	22 (28.21%)^a^	0.02

BMI: body mass index. Data are presented as mean ± standard error of the mean, n (%), or median (range).

Table 1 shows the patient characteristics of the three groups. The oxytocin administration rates in the control and ropivacaine groups were significantly lower than that in the levobupivacaine group (P=0.02).

^a^P<0.05 vs levobupivacaine group.

**Table 2:** Maternal and fetal outcomes.

	**Control**	**Levobupivacaine**	**Ropivacaine**	**P value**
	**n = 69**	**n = 66**	**n = 78**	
**Cesarean, n (**%**)**	4 (5.80%)	10 (15.15%)	7 (8.97%)	0.18
**Instrumental, n (**%**)**	7 (10.15%)	5 (7.58%)	5 (6.41%)	0.70
**Neonatal birth weight (g)**	3148.33 ± 33.81	3215.53 ± 39.58	3272.31 ± 44.90	0.08
**Duration of 1st stage of labor (min)**	562.00 ± 25.06^b^	677.14 ± 32.36	590.63 ± 25.41^b^	0.01
**Postpartum hemorrhage (ml)**	252.46 ± 10.09	247.73 ± 8.93	245.38 ± 10.99	0.88
**1 minute Apgar **	9(8-10)	9(7-10)	9(6-10)^a^	<0.001
**5 minute Apgar **	9(8-10)	9(8-10)	9(9-10)^a^	<0.001
**10 minute Apgar **	9(9-10)	9(8-10)	9(8-10)	0.98
**Meconium stained amniotic fluid, n (**%**)**	9 (13.04%)	12 (18.18%)	18 (23.38%)	0.29

Data are presented as mean ± standard error of the mean, n (%), or median (range).

Table 2 shows maternal and fetal outcomes in the three groups. The episiotomy rate (P=0.001) and Apgar scores at 1 and 5 min (P<0.001) in the ropivacaine group were significantly higher than those in the levobupivacaine and control groups. Both the control and ropivacaine groups had a shorter duration of first stage of labor than that described in the levobupivacaine group (P=0.01).

^a^P<0.001 vs control or levobupivacaine group.

^b^P<0.05 vs levobupivacaine group.

**Table 3:** Uterine EMG activities in three groups.

	**Control**	**Levobupivacaine **	**Ropivacaine **	**P value**
	**n = 69**	**n= 66**	**n= 78**	
**Duration (s)**	44.92 ± 2.15	52.04 ± 3.30	49.35 ± 1.46	0.66
**Number of bursts (n)**	3.65 ± 0.13	3.64 ± 0.14	3.49 ± 0.07	0.05
**RMS (mV)**	0.09 ± 0.01^a^	0.05 ± 0.004	0.06 ± 0.01	<0.001
**Power (nV** ^**2**^ **)**	7.69 ± 1.37^b^	3.42 ± 0.59	6.19 ± 1.31^b^	0.005
**Peak frequency (Hz) **	0.43 ± 0.01	0.42 ± 0.01	0.45 ± 0.01^c^	0.03

RMS: root mean square. Data are presented as mean ± standard error of the mean.

Table 3 shows uterine EMG activities of the three groups. The RMS (P<0.001) in the control group was significantly higher than those in the levobupivacaine and ropivacaine groups. Both the control and ropivacaine groups had a higher power than the levobupivacaine group (P=0.005). There were no significant differences in the duration or number of bursts among the groups (P>0.05).

^a^P<0.001 vs levobupivacaine or ropivacaine group.

^b^P<0.05 vs levobupivacaine group.

^c^P<0.05 vs control or levobupivacaine group.

**Table 4:** Uterine EMG activities in the ropivacaine group.

	**30 min**	**60 min**	**120 min**	**P value**
	**n = 26**	**n = 27**	**n = 25**	
**Duration (s)**	46.29±2.37	49.65±2.58	52.22±2.56	0.26
**Number of bursts (n)**	3.31±0.13	3.48±0.11	3.68±0.11	0.09
**RMS (mV)**	0.06±0.01	0.06±0.01	0.06±0.01	0.97
**Power (nV** ^**2**^ **)**	5.95±1.71	5.42±1.15	7.26±3.52	0.85
**Peak frequency (Hz)**	0.43±0.01	0.47±0.01	0.44±0.01	0.06

RMS: root mean square. Data are presented as mean ± standard error of the mean.

## Data Availability

The data used to support the findings of this study are included within the article.
